# Buffalo Medical and Surgical Association

**Published:** 1891-09

**Authors:** W. S. Renner

**Affiliations:** Secretary


### BUFFALO MEDICAL AND SURGICAL ASSOCIATION.

Reported by W. S. RENNER, M. D., Secretary.

REGULAR meeting held Tuesday, July 7, 1891, in the parlors of the Hotel Iroquois.

Dr. EDWARD CLARK in the chair. Dr. A. L. BENEDICT read a paper on the Treatment of Chronic Dyspepsia, and Dr. ALBERT E. PERSONS read a paper on Disorders of Nutrition.

DISCUSSION OF BOTH PAPERS.

Dr. HAYD said that it was not surprising that we had so much dyspepsia in this country, owing to our habits of life. Washing out the stomach had proven a great aid in the diagnosis and treatment of diseases of the stomach, in fact it is the only treatment in chronic dyspepsia ; it carries away all irritating substances, stimulates the muscular walls, and, perhaps, even regenerates some of the tubules. He related the history of a case : A woman weighing eighty-six pounds, with dyspepsia, but no organic disease of stomach, (in this case he found traces of hydrochloric acid in the contents of the stomach,) had increased in weight under this treatment until now she weighs 186 pounds. He does not believe that medicines are of any use in these cases. You must first remove the fermentary elements. He has used both the faradic and galvanic currents in dilatation of the stomach, with good results. He believed that the belching and acidity, in many cases, is not due to stomach disease, *per se*, but is of reflex origin.

Dr. HARTWIG said that it was necessary to examine into the habits of the patient for the cause of indigestion in many cases. To get contents of the stomach for examination, he is in the habit of letting the patient swallow a small piece of sponge attached to a string and enclosed in a capsule. The sponge is drawn out after the capsule has dissolved. The contents are then squeezed out of the sponge and examined for hydrochloric acid.

Dr. BARTLETT had found the test for hydrochloric acid of value in diagnosticating a case as one of ulcer, and not cancer, of the stomach.

Dr. E. H. LONG thought the term dyspepsia should be dispensed with, and *indigestion* used instead, except when the gastric disturbance is due to disease of some other organ ; in which case the cause should be stated.

Dr. CLARK thought that physicians " drug " their patients too much for stomach troubles. Much trouble was due, in children, to the formation of tyrotoxicon in the milk. In some cases milk is an absolute poison to children.

Dr. HAYD reported a case of paresis of syphilitic origin, greatly benefited by very large doses of iodide of potash. The patient is now under the care of Dr. Loomis, of New York, who is giving her 480 grains of iodide daily.

Dr. HARTWIG reported a case of syphilitic laryngitis.

Meeting adjourned at 10.30 P. M.

